# Regulation of Tyrosine Phosphatase STEP61 by Protein Kinase A during Motor Skill Learning in Mice

**DOI:** 10.1371/journal.pone.0086988

**Published:** 2014-01-23

**Authors:** Laure Chagniel, Yan Bergeron, Geneviève Bureau, Guy Massicotte, Michel Cyr

**Affiliations:** Groupe de recherche en Neurosciences, Département de biologie médicale, Université du Québec à Trois-Rivières, Trois-Rivières, Québec, Canada; Karolinska Inst, Sweden

## Abstract

Recently, striatal-enriched protein tyrosine phosphatase (STEP) and its upstream regulator protein kinase A (PKA) have been suspected to play a role in the intracellular mechanisms of fear conditioning and spatial memory. However, whether they contribute to the learning and memory of motor skills is totally unknown. In this study, we have investigated the role of STEP and PKA activities during motor skill learning associated with the accelerating rotarod task. We observed that learning the rotarod task differentially modulated the levels of phosphorylated STEP61 at serine 221, a site directly regulated by PKA, in the hippocampus, motor cortex and striatum. In a second set of experiments, we have pharmacologically inhibited PKA by the injection of Rp-cAMPS directly into the dorsal striatum of mice before rotarod trainings. PKA phosphorylation of STEP prevents the dephosphorylation of STEP substrates, whereas inhibition of PKA promotes STEP activity. Striatal PKA inhibitions dose-dependently impaired mice performances on the accelerating rotarod task. General motor abilities testing revealed an intact motor control in mice treated with 5 and 20 µg of Rp-cAMPS, but not at the highest dose of 40 µg. This suggested that motor learning was selectively affected by PKA inhibition at lower doses. Most notably, striatal inhibition of PKA reduced the levels of phosphorylated STEP61 at serine 221. Our data support that inactivation of STEP61 by the PKA activity is part of the molecular process associated with motor skill learning.

## Introduction

Motor skill learning refers to the process by which a complex movement sequence is encoded in the brain. Once memorised, a common task is performed without effort and is quickly executed despite a prolonged period of time without practice. The learning process associated with the acquisition of motor skills involves two stages (fast and slow learning stages) and brain areas including striatum, cerebellum, hippocampus and motor cortices regions [1], [2]. Undeniably, motor learning processes are mediated by specific brain molecular changes. For instance, it has been demonstrated that motor skill learning induces novel expressions of important genes and proteins in the striatum and motor cortex [3]–[7]. However, only few studies have investigated motor learning at the level of proteins activity [6], [8]–[11]. Additional investigations are definitely required to understand the molecular determinant of this type of learning.

Striatal-enriched protein tyrosine phosphatase of 61 kDa (STEP61) is brain-specific. It is expressed in brain area involved in motor learning that include the striatum, hippocampus and cortex [12]–[14]. It has been demonstrated *in vitro* that STEP61 activity is negatively regulated by protein kinase A (PKA). For instance, it is well accepted that PKA phosphorylation at the conserved serine residue 221 (Ser221) of STEP61 induced a reduction in STEP61 activity [15]. PKA is a ubiquitously expressed kinase that has been documented to play an important role in the synaptic plasticity of learning and memory [16], [17]. Behavioral experiments demonstrate that intra-amygdala or intra-hippocampal pharmacological inhibition of PKA interfere respectively with fear conditioning consolidation [18] and spatial memory in a Morris water maze [19]. Transgenic mice expressing a dominant negative form of the regulatory subunit of PKA exhibit impaired spatial memory and long term memory for contextual fear conditioning [20], [21]. It has also been demonstrated that STEP contributes to fear and spatial memories through the regulation of neuronal signaling [22]–[24]. Although STEP and PKA are implicated in spatial memory and fear conditioning consolidation; their role in motor skill learning is still uncovered.

The present study investigates the involvement of STEP and its relationship with PKA in motor skill learning processes associated with the accelerating rotarod task in mice. Our finding reveals that the levels of phosphorylated STEP61 are differentially modulated in the brain of mice during motor skill learning. Furthermore, in the dorsal striatum structure, we demonstrate that PKA activity influences the phosphorylation levels of STEP61 at Ser221 residue, and directly contributes to the acquisition of a complex motor task.

## Materials and Methods

### Ethics Statement

All experimental procedures were reviewed and approved by the UQTR Committee on Animal Care (Protocol Number: 2012-MIC.17), and were in accordance with ethical standards of the Canadian Council on Animal Care. Full efforts were made to minimize suffering and discomfort of the animals.

### Animals

Male C57bl/6j mice (12 weeks-old) were obtained from Charles River (St-Constant, QC, Canada). Mice were housed in a climate-controlled room (14-h light/10-h dark cycle) with food and water available *ad libitum*.

### Experimental Design

**Figure 1:**
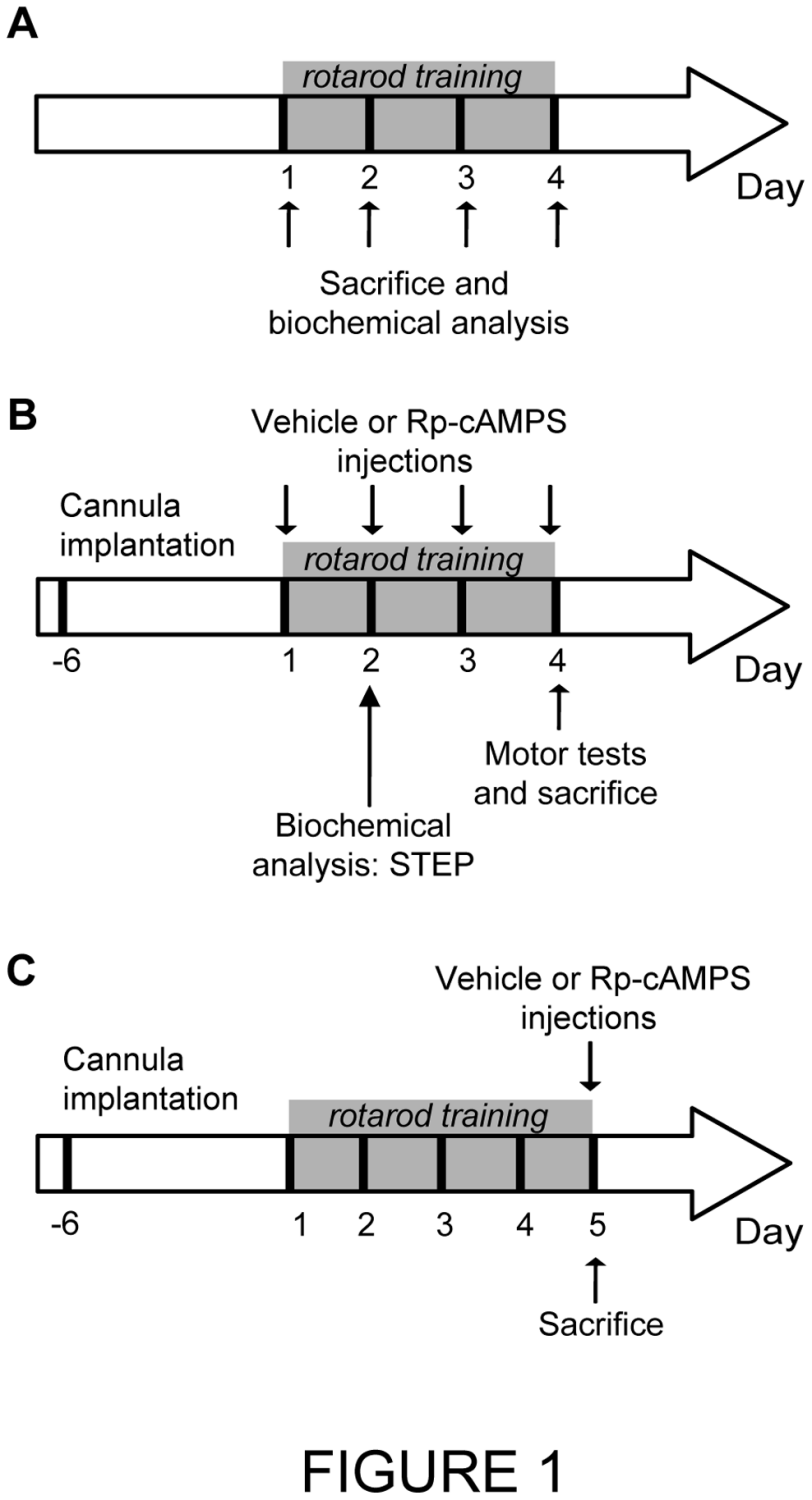
Experimental design. (**A**) Analysis of STEP expression during motor skill learning. Drug naïve mice were trained on the accelerating rotarod and sacrificed immediately after the end of the last trial at each day of training (day 1, 2, 3 and 4). Levels of total and phosphorylated STEP proteins in selected brain regions were analyzed by western blot techniques. (**B**) Role of PKA in motor learning. A guide cannula was implanted into the dorsal striatum of mice 7 days before rotarod training. Rp-cAMPS or vehicle were injected directly into the dorsal striatum of mice before the first trial at each rotarod training day (day 1, 2, 3 and 4). At the fourth day of training, mice were also tested for their motor abilities. Another cohort of mice received Rp-cAMPS or vehicle before the first trial at each rotarod training day. Mice were sacrificed at the second training day to performed western blot experiments. (**C**) Role of PKA activity in the motor coordination requested during rotarod testing. A guide cannula was implanted into the dorsal striatum of mice 7 days before rotarod training. Mice were trained during four consecutive days and Rp-cAMPS or vehicle was injected before the first trial at the fifth training day.

In order to determine the level of STEP phosphorylation during motor skill learning a cohort of drug naïve mice were trained on the accelerating rotarod and sacrificed immediately after the end of the last trial at each day of training (**Figure 1A**). Brain of trained (*n* = 4 mice/training day) and untrained mice (*n* = 4) were removed, carefully dissected, rapidly frozen on dried ice and preserved at −80°C until western blot analysis To examine the role of PKA, Rp-cAMPS or vehicle (saline) were injected directly into both hemisphere of the dorsal striatum, 15 minutes prior to the first trial of each training days, in an independent cohort of mice (**Figure 1B**). The following group of mice were included: (1) vehicle-treated mice (control, *n* = 7), (2) Rp-cAMPS-treated mice at dose 5 µg/side (*n* = 4), (3) Rp-cAMPS-treated mice at dose 20 µg/side (*n* = 4) and (4) Rp-cAMPS-treated mice at dose 40 µg/side (*n* = 4). At the last rotarod training session, the pole, wire suspension and stepping tests were performed in all mice to test their motor abilities. To determine whether PKA mediate STEP phosphorylation in the striatum of trained mice, levels of STEP phosphorylation after Rp-cAMPS injections were measured in a third independent cohort of mice after two days of training. Mice received intrastriatal injections of vehicle (*n* = 4) or 20 µg/side of Rp-cAMPS (*n* = 4), 15 min prior to the first trial of each rotarod training day. Mice were sacrificed immediately after the last trial of the second training day. Brain were removed, rapidly frozen on dried ice and preserved at −80°C. A fourth cohort of mice was used to assess motor coordination on the rotarod following PKA inhibition (**Figure 1C**). Drug-naïve mice were trained on the accelerating rotarod during four consecutive days where performances reached a plateau, meaning the mice had fully learned the task. The day after, at their fifth day of training, mice received vehicle (*n* = 4), 20 µg/side (*n* = 4) or 40 µg/side (*n* = 4) of Rp-cAMPS directly into the dorsal striatum, 15 minutes prior to the first rotarod trial.

### Pharmacological Treatments

The PKA inhibitor Rp-cAMPS was purchased from Tocris Bioscience (Bristol, United Kingdom). Rp-cAMPS or vehicle (saline) was administered bilaterally into the dorsal striatum, 15 min before the first rotarod trial. Rp-cAMPS at the dose of 5, 20 or 40 µg in 1 µl/site (dissolved in saline) and vehicle were injected by microinjection at a constant rate of 0.5 µl/min. Injections were performed under the control of a micro injector pump (Harvard Apparatus, Holliston, MA, USA). Drug doses were chosen based on previous studies [25], [26]. For the microinjections, a bilateral 26 gauge guide cannula (Plastics One, Roanoke, VA, USA) was implanted in mice under anesthesia with isoflurane, 7 days before the beginning of treatments. The precise localisation of the gauge guide cannula in the dorsal striatum was made using a stereotaxic apparatus. The coordinates were AP: +0.86 mm; ML: ±1.50 mm; DV: −3.25 mm relative to Bregma, according to the atlas of Paxinos and Franklin [27].

### Rotarod Test

The accelerating rotarod apparatus (AccuScan Instruments, Columbus, OH, USA) was consisting of a suspended rod that accelerates at a constant rate, from 4 to 40 rpm in 300 s. At each day of training, mice were trained on the rotarod throughout a session of 10 trials. A trial ends when the mouse falls off the rod or after reaching 300 s. Time was recorded for each trial. A resting time of 180 s was allowed between each trial.

### Motor Ability Tests

General motor behaviour of mice was evaluated using the wire suspension, pole and stepping tests as we described previously [28], [29]. At the fourth day of rotarod training, animals were pre-trained three times to ensure the tasks were fully learned. Afterward, Rp-cAMPS or vehicle solutions were injected. 15 min later, motor and rotarod tests were performed (**Figure 1B**). Briefly, the wire suspension test consisted to hang the animal with its paws on the middle of a wire fixed horizontally between two platforms (length: 80 cm, height: 25 cm). The time needed to reach one platform was recorded. For the pole test, mice were placed on the top of a pole (length: 50 cm, diameter: 1.5 cm), and the time taken to turn down and reach the floor was recorded. The maximal time allowed for the wire suspension and pole test was set at 120 s. The stepping test consists to lift up the animal’s hind legs by pulling up the tail, leaving only the forepaws on the table. Then, the experimenter pulled the animal backward by the tail (1 m in 5–6 s), until the other edge of the table was reached. Each trial was recorded using a video camera, and for each the number of adjusting steps for both forepaws was calculated.

### Western Blotting

Immediately after each day of training, brains of drug naïve mice were removed and anterior cortex, striatum and hippocampus were carefully dissected, rapidly frozen on dried ice and preserved at −80°C until protein extraction. To measure STEP expression after Rp-cAMPS injection, a micro punch tissue was performed in both hemispheres of the striatum at the injection site [30]. To this end, a 1 mm thick slice of frozen brain containing the striatum (Bregma from 1.10 mm to 0.10 mm) was cut using Leica CM3050 cryostat (Leica Biosystems, Concord, ON, Canada). Bilateral punches were then collected from the dorsal striatum using a 2 mm punch (Stoelting, Wood Dale, IL, USA). All tissue samples were homogenized in RIPA buffer containing protease and phosphatase inhibitors cocktails (Roche, Indianapolis, IN, USA). Protein concentrations were quantified by Bradford assay (Bio-Rad, Hercules, CA, USA). 40 µg of every protein sample was loaded on 10% SDS-PAGE gel electrophoresis and transferred on nitrocellulose membranes. The membranes were blocked in 5% BSA/TBS-Tween 0.1% for 1 hour at room temperature and incubated overnight at 4°C with the primary antibodies. The following primary antibodies were used: rabbit polyclonal antibody against phospho-STEP61 (Ser221) (1∶1000; Millipore, Billerica, MA, USA), mouse monoclonal antibody against STEP (1∶2000; Upstate-Millipore), rabbit polyclonal antibody against phospho-CREB Ser133 (1∶1000; Cell Signaling Technology, Beverly, MA, USA) and rabbit polyclonal antibody against CREB (1∶1000; Cell Signaling). Specificity of phospho-STEP and STEP antibodies was examined (**Figure S1A, B**). The membranes were washed in TBS-Tween 0.1% and incubated with appropriate horseradish peroxidise conjugated secondary antibody (1∶5000; Cell Signaling Technology, Beverly, MA, USA). Mouse monoclonal antibody against GAPDH (1∶10000; Abcam, Cambridge, MA, USA) served as a loading control. Chemiluminescence reactions (SuperSignal West Femto chemiluminescence kit, Pierce Chemical Co., Rockford, IL, USA) were utilized to visualize proteins. Densitometric analyses were performed using the Vision work LS software (UVP Bioimaging, Upland, CA, USA) and data were expressed as relative optical density.

### Statistical Analysis

Statistical analyses were performed using the GraphPad Prism software (version 5.0, Graph Pad Software, San Diego, CA, USA). Data were subjected, as appropriate, to an unpaired t-test, one-way ANOVA followed by the Tukey *post hoc* test. Data were reported as the mean ± S.E.M. Statistical significance was set at *p*<0.05.

## Results

### Modulation of STEP61 Phosphorylation during Rotarod Training

**Figure 2:**
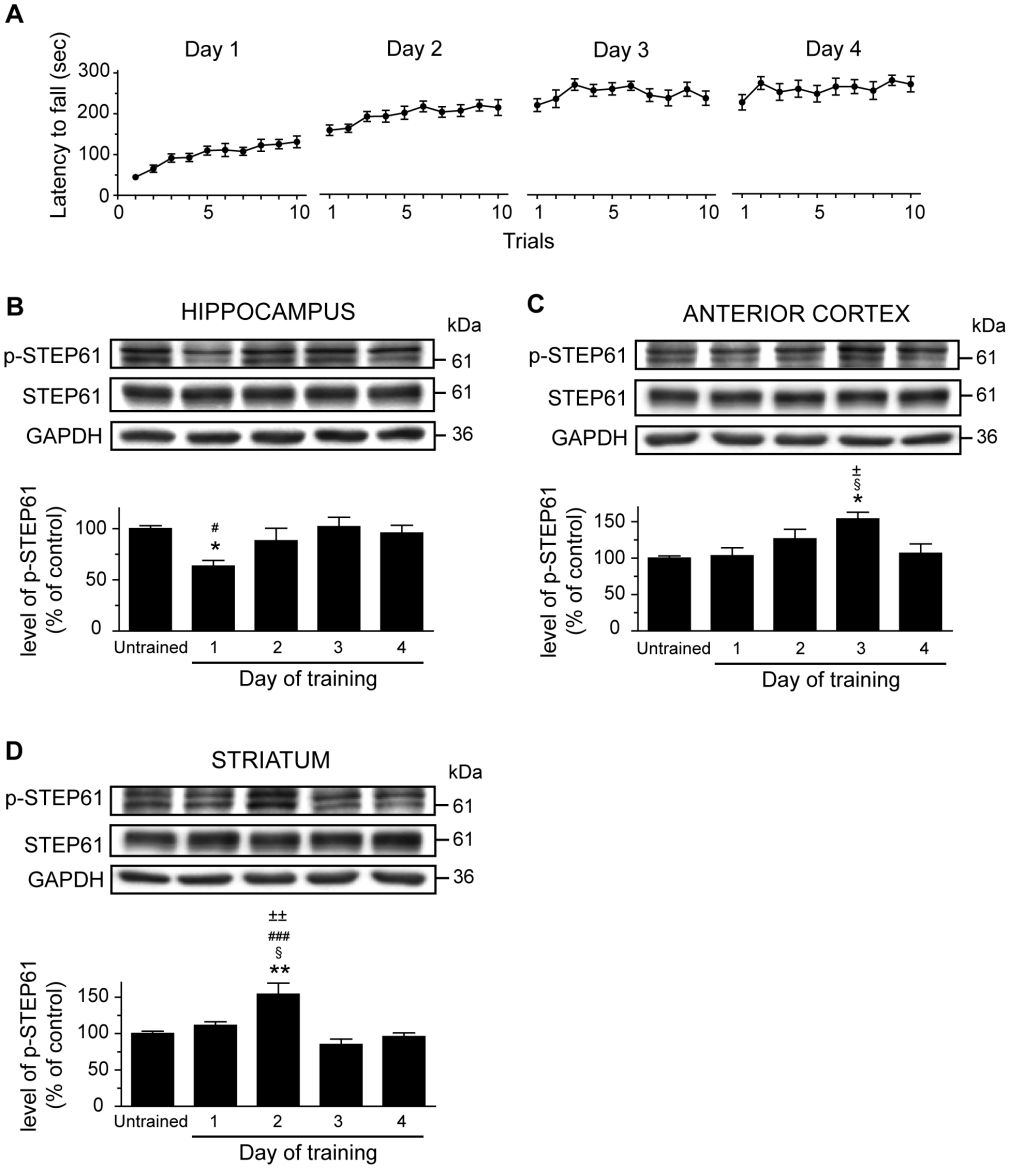
Levels of phosphorylated STEP61 in the brain of mice during motor learning. (**A**) Drug-naïve mice were trained on the accelerating rotarod during 4 consecutive days and sacrificed at the end of each training day. Protein levels were evaluated by western blot. Proteins were extracted from selected brain regions of untrained or trained mice. Protein levels of phosphorylated STEP61 at Ser221, total STEP61 as well as GAPDH were measured in (**B**) hippocampus, (**C**) anterior cortex and (**D**) striatum. Data represent the mean of p-STEP61 relative optical density (expressed as a percentage of control values) ± S.E.M. Values are expressed relative to total STEP and are from triplicate experiments/animal, *n* = 4 mice/group. *p<0.05, **p<0.01 vs. untrained mice; ^§^ *p*<0.05 vs day 1; ^#^ *p*<0.05, ^###^ *p*<0.001 vs. day 3; ^±^ *p*<0.05, ^±±^ *p*<0.01 vs. day 4.

As we and other have previously documented, drug naïve mice rapidly improved their performances on the rotarod task at the first day of training whereas at the second day, their scores improved slowly and reached a plateau at the third day (**Figure 2A**) [6]. In these trained mice, we investigated whether motor skill learning influenced STEP phosphorylation at Ser221 for STEP61. To this end, levels of phosphorylated STEP61 at Ser221 were measured, using western blot technique, at each training day (day 1, 2, 3 and 4). The brain regions considered were chosen based on their known implication in motor learning as well as the presence of STEP expression [12]. Note that the selectivity of STEP antibodies were evaluated (**Figure S1A**). In the hippocampus, we observed a decrease of 37% in p-STEP61 levels at the first day of training compared to untrained control mice. (**Figure 2B**; *F*_(4;19)_ = 3.689; *P<*0.05; one-way ANOVA followed by the Tukey post-hoc). In the anterior cortex, levels of p-STEP61 were significantly increased by 54% at the third day (**Figure 2C**; *F*_(4;19)_ = 4.597; *P<*0.05). In the striatum, a significant increase of 54% in the levels of p-STEP61 were observed at second day of training compared to untrained control mice (**Figure 2D**; *F*_(4;19)_ = 10.150; *P<*0.001) Notably, levels of total STEP61 protein were unchanged during rotarod learning.

### Striatal PKA Inhibition Impaired Rotarod Learning

**Figure 3:**
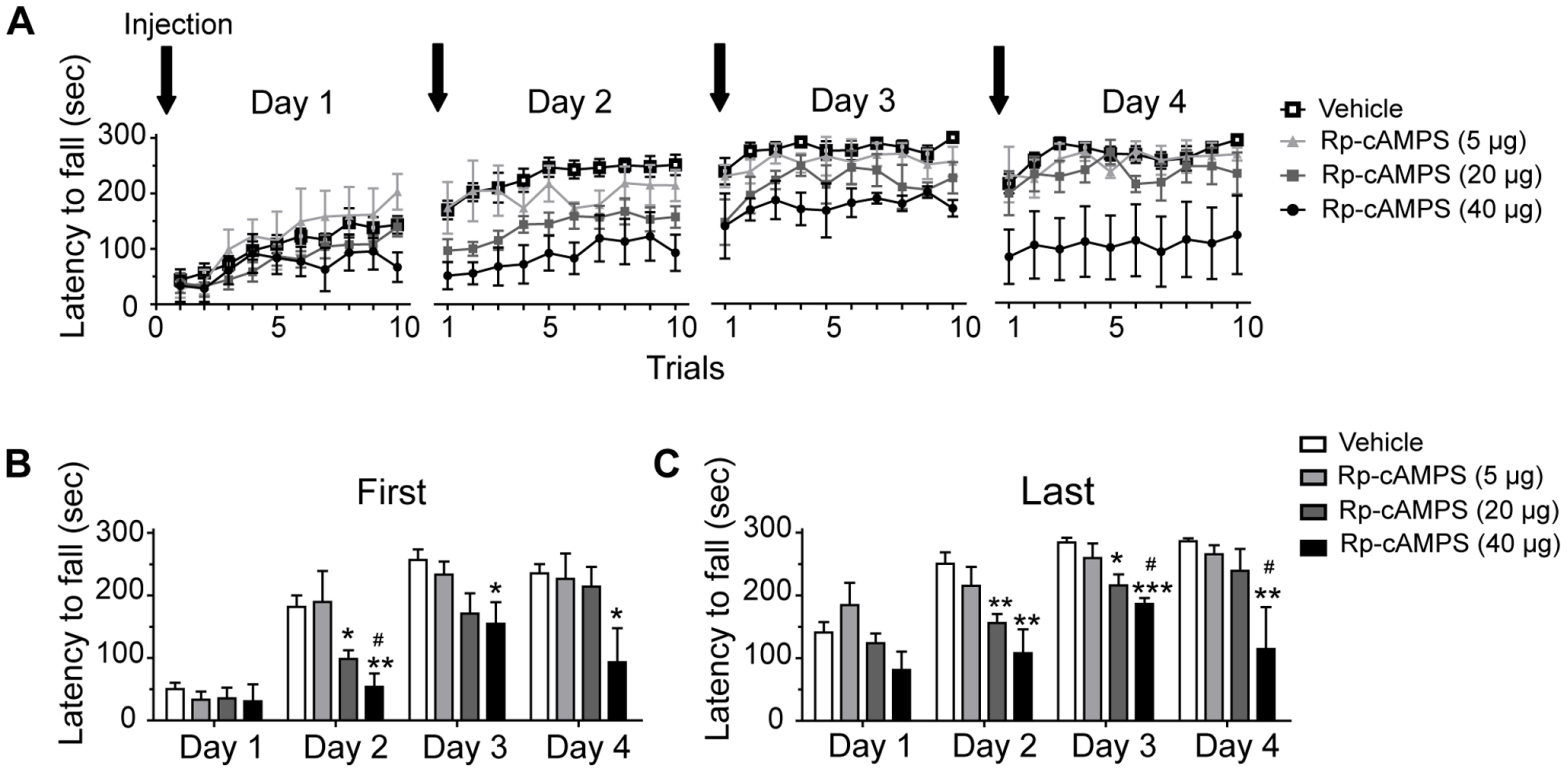
Effect of intrastriatal inhibition of PKA in mice during rotarod training. (**A**) Time spent on the rod of the accelerating rotarod for every trial completed at days 1, 2, 3 and 4. Mice were treated directly into the dorsal striatum, 15 min before each training day, with vehicle (saline) or Rp-cAMPS at doses: 5, 20 or 40 µg/side. Data represent the mean of latency to fall per trial expressed for every training day ± S.E.M. Lower panels represent the average scores of (**B**) the two first trials and (**C**) the two last trials of each training day. Values represent the average mean latency to fall expressed in seconds ± S.E.M. *n* = 4 to 11 mice/group. *p<0.05, **p<0.01 ***p<0.001 vs. respective vehicle-treated mice; ^#^ *p*<0.05 vs. mice treated with 5 µg/side.

It has been demonstrated that STEP61 is phosphorylated by PKA at Ser221 in striatal homogenates or in striatal slices [15]. Our result that phosphorylation of STEP61 at Ser221 is augmented in the striatum during rotarod training (**Figure 2C**) suggests an increased activation of PKA in this structure. To investigate whether striatal PKA activity play a role in motor skill learning, we injected directly into the dorsal striatum of mice a competitive inhibitor of PKA, Rp-cAMPS, 15 min before each accelerating rotarod training day. Three different doses of Rp-cAMPS were used: 5, 20 and 40 µg/side. We observed that Rp-cAMPS dose-dependently impaired rotarod performances across training days (**Figure 3A**). Statistical analysis using one-way ANOVA followed by the post hoc Tukey test was performed on the average of the two first trials (**Figure 3B**) and two last trials (**Figure 3C**) of every training session. Similar rotarod performances were observed in mice treated with either 5 µg/side of Rp-cAMPS or vehicle (**Figure** **3B, C**). In contrast, mice treated with 20 or 40 µg/side of Rp-cAMPS demonstrated lower performances across training day compared to vehicle-treated mice (**Figure 3B**: day 1: *F*_(3;26)_ = 0.358, *P*>0.05, day 2: *F*_(3;26)_ = 8.283, *P*<0.001, day 3: *F*_(3;18)_ = 3.991, *P*<0.05, day 4: *F*_(3;18)_ = 3.795, *P*<0.05; **Figure 3C**: day 1: *F*_(3;26)_ = 2.495, *P*>0.05, day 2: *F*_(3;26)_ = 7.882, *P*<0.001, day 3: *F*_(3;18)_ = 10.60, *P*<0.001, day 4: *F*_(3;18)_ = 5.600, *P*<0.01). Inhibition of PKA with 20 µg/side of Rp-cAMPS resulted in reduced performances only at the second and third training days whereas the dose of 40 µg/side was associated with robust impaired performances at day 2, 3 and 4.

### Integrity of Motor Abilities during Striatal PKA Inhibition

**Figure 4:**
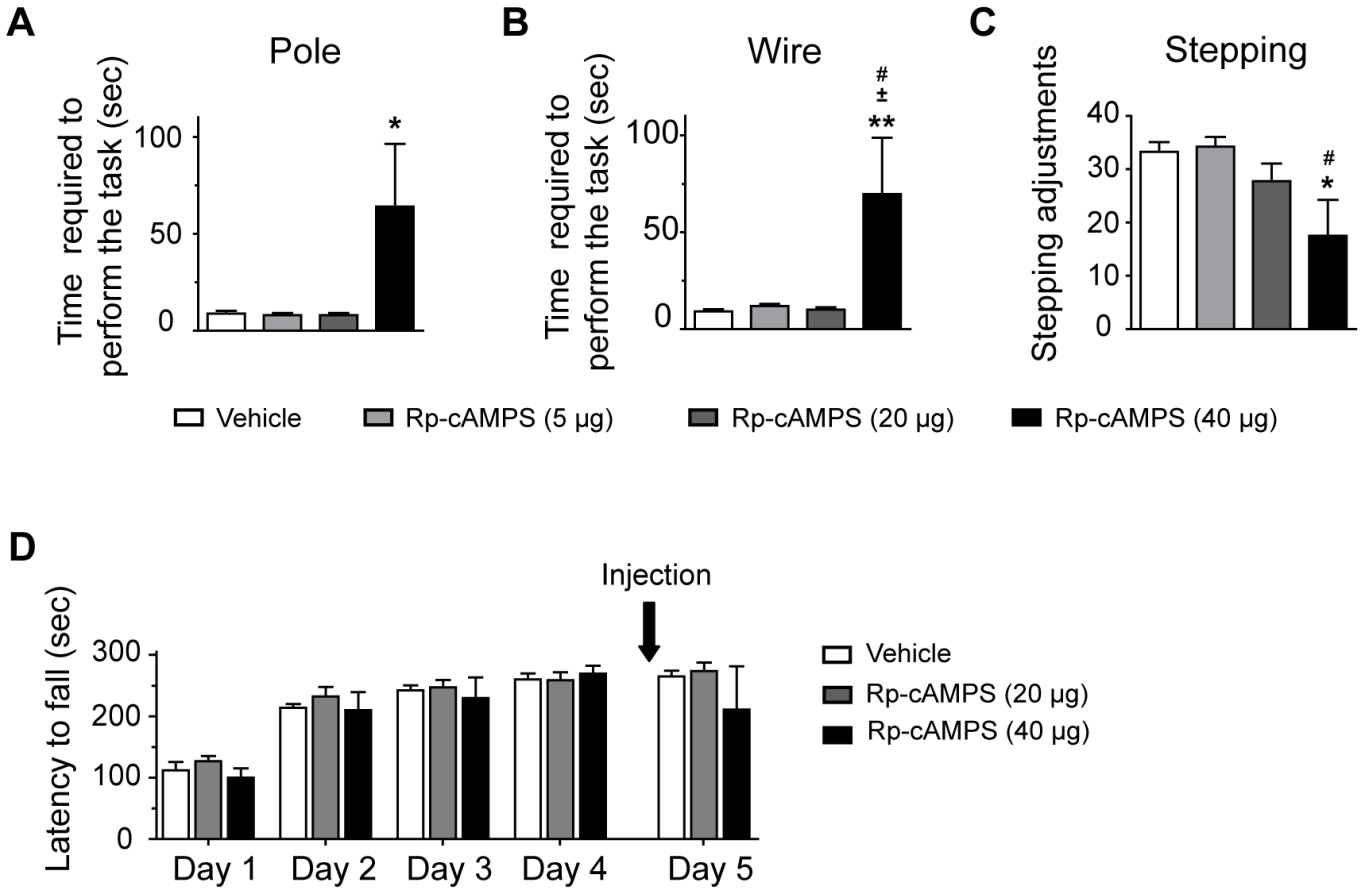
Motor abilities in mice with intrastriatal inhibition of PKA. (**A**) Pole, (**B**) wire suspension and (**C**) stepping tests were performed to assess motor abilities in saline (vehicle)-treated mice as well as mice treated with 5, 20, or 40 µg/side of Rp-cAMPS into the dorsal striatum. Data represents the mean of time require to perform the test (pole and wire suspension test) and the mean of numbers of adjusting steps (stepping test) ± S.E.M. *n* = 4 to 7 mice/group. *p<0.05, **p<0.01 vs. respective vehicle-treated mice; ^#^ *p*<0.05 vs. mice treated with 5 µg/side; ^±^ *p*<0.05 vs. mice treated with 20 µg/side. (**D**) Evaluation of motor coordination on the accelerating rotarod. Drug-naïve mice were trained on the accelerating rotarod during 4 consecutive days and treated into the dorsal striatum at the fifth day with vehicle (saline), 20 or 40 µg/side Rp-cAMPS. The total average latency to fall is shown at every training day in mice treated with vehicle or Rp-cAMPS. Data represent the means of all trials per training day ± S.E.M. *n* = 4 mice/group.

To verify that the effects of PKA inhibition on rotarod performances are not due to impaired general motor abilities of mice, we performed three well recognized motor execution tests: the pole, wire suspension and stepping tests. The pole test estimated bradykinesia and motor coordination; the wire suspension test analyzed the coordination and muscle strength of mice and the stepping test evaluated akinesia [31]–[34]. In order to remove the learning variables associated with these tests, mice were pre-trained. We tested motor capacities of mice after inhibition of PKA at the fourth day. No significant difference between vehicle-treated mice and mice treated with 5 or 20 µg/side of Rp-cAMPS was observed on these three tests (**Figure 4A–C**; ANOVA). Interestingly, motor capacity of mice treated with 40 µg/side was impaired in all tests (pole test: *F*_(3;18)_ = 3.944, *P*<0.05, wire suspension test: *F*_(3;18)_ = 5.574, *P*<0.01, stepping test: *F*_(3;18)_ = 4.174, *P*<0.05). Moreover, we investigated directly the effect of PKA inhibition on rotarod motor coordination. To this end, drug naïve mice were trained on the accelerating rotarod during 4 consecutive days. At the fifth training day, we injected 20 or 40 µg/side of Rp-cAMPS in the dorsal striatum and observed that PKA inhibition at both doses had no statistically significant effect on rotarod performances (**Figure 4D**, ANOVA; day 5: *F*_(2;11)_ = 0.658; *P>*0.05).

### PKA Activity Altered Striatal STEP61 Phosphorylation

**Figure 5:**
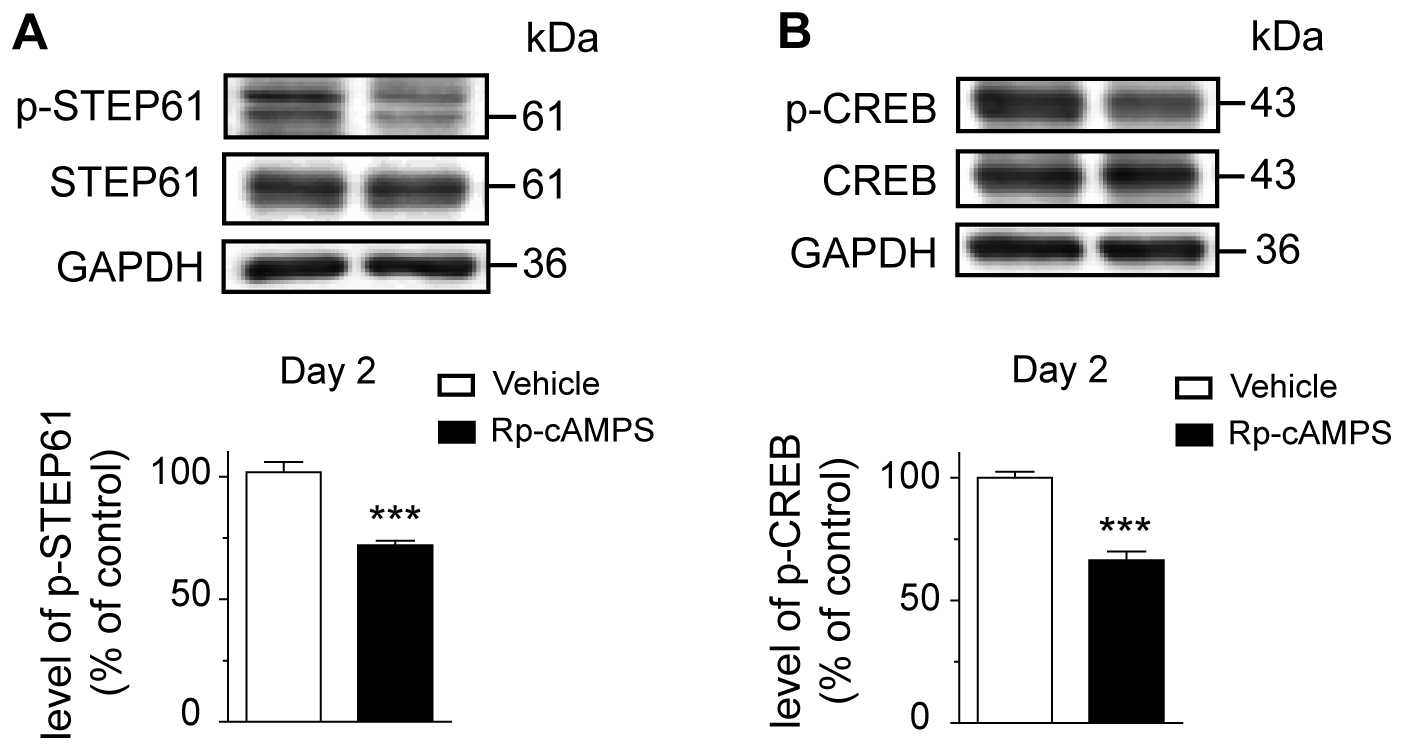
Levels of phosphorylated STEP61 and CREB in mice with intrastriatal inhibition of PKA. (**A**) Levels of phosphorylated STEP61 at Ser221 and (**B**) phosphorylated CREB at Ser133 were evaluated in the dorsal striatum of trained mice injected with vehicle (saline) or Rp-cAMPS at the second day of training. Data represent the mean of relative optical density of phosphorylated STEP61 and phosphorylated CREB (expressed as a percentage of control values) ± S.E.M. Values are respectively expressed relative to total STEP and total CREB. Values are from triplicate experiments/animal, *n* = 4 mice/group. ***p<0.001 vs. vehicle-treated mice.

Next, we investigated whether STEP61 phosphorylation in the striatum of trained mice is PKA dependant. An independent cohort of mice was trained on the accelerating rotarod and received 20 µg/side of Rp-cAMPS, 15 minutes prior the first trial of each training day. Since we noticed an increase in striatal p-STEP61 level at the second day of learning, mice were sacrificed at the end of the last trial of day 2. Levels of p-STEP61 in the dorsal striatum were measured using the western blot technique. We demonstrated that intrastriatal PKA inhibition decreased by 30% the levels of p-STEP61 when compared to vehicle-treated mice (**Figure 5A**, Unpaired t test, *P<*0.001). Moreover, inhibition of PKA was confirmed by quantification of the phosphorylation of a known substrate of PKA, the transcription factor cAMP response element-binding protein (CREB), at Serine 133 [35]. Intrastriatal inhibition of PKA decreased by 35% the levels of p-CREB (**Figure 5B**, Unpaired t test, *P<*0.001).

## Discussion

In this present study, we have investigated whether the activity of 61 kDa STEP isoform is modulated in the brain of mice that are learning a complex motor task, and whether PKA play a role in these processes. To assess the learning of a complex motor skill, we select the accelerating rotarod test, among many other tasks such as chaining of motor sequences, visuomotor skill acquisition, instrumental lever-pushing and serial reaction-time tests. The benefit of this test, in contrast to the other tasks, is its association with clear dependent-adaptive responses memory. This test can be arbitrary divided into two stages in parallel to the general pattern of memory encoding that include: an early, fast learning stage, in which larger improvement of performance can be observe within a single training session (day 1) and a later, slow learning stage, in which incremental gains in performance can be observed across several sessions (day 2, 3 and 4). Moreover, it allows the investigation of motor skill learning in the absence of the associative and working memory components of other motor learning tasks. Motor abilities are distinguished from motor learning by assessing general motor capacities through the stepping, wire suspension, and pole tests.

Our findings propose that the learning processes associated with rotarod learning involve STEP61 activity. We demonstrate that at an early phase of learning, at the end of the first training day, levels of phosphorylated STEP61 are decreased in the hippocampus of trained mice, whereas they are comparable to untrained mice during later phases, after the second, third and fourth day of training. In the hippocampus, previous studies demonstrate that STEP deletion facilitated hippocampal-dependent learning [23]. Our data are therefore in contradiction with this study based on the current proposed molecular model that STEP activation acts as a tonic brake on synaptic transmission. Our observation of decreased levels of phosphorylated STEP would be associated with enhanced STEP activity, and therefore a reduction in the synaptic strength. One possibility is that STEP is behaving as an auto-regulator on this first day of training, to turn down and compensates the enhanced activity of proteins implicated in synaptic reorganisation processes. This may explain why these phosphorylation levels return to baseline levels in the following sessions (day 2, 3 and 4). We have indeed previously observed that markers of adaptive response to synaptic activation for newly learned events are activated in the hippocampus after the first day of learning, and return to basal levels in the following days of learning [6]. This finding indicates that the hippocampus might be engaged in the early learning phase. In contrast, in the anterior cortex and striatum, an increase in the levels of phosphorylated STEP is observed during later phases of motor skill training. We indeed noticed a punctual surge in STEP phosphorylation at the second day in the striatum and at the third day in the anterior cortex. The second and third day of our rotarod training paradigms are reflecting the later phases of learning. The anterior cortex and striatum seems to be engaged in these periods specifically and this is in agreement with previous studies. It has been documented that both structures are further engaged when the range of gain of performance is much lower [2], [6], [36]. Altogether, our data demonstrated that STEP activity during motor skill learning is neuroanatomically defined and depends of the learning phases. Further experiments are definitively needed to understand this regional brain specificity of STEP activity during motor skill learning. However, although STEP has been linked previously to certain learning behaviours [22]–[24], to our knowledge, we are the first to report a brain differential modulation of STEP activity during motor skill learning.

Levels of phosphorylated STEP61 are modulated during rotarod learning at the Ser221 residue. STEP61 is known to be phosphorylated at this serine residue *in vitro*, in brain slices and striatal homogenates, by PKA activity [15]. Our data confirms *in vivo*, for the first time, a direct relationship between PKA activation and phosphorylation of STEP Ser221 residue. We indeed demonstrate that inhibition of PKA into the dorsal striatum of mice decreases phosphorylation levels of STEP61 at Ser221. Interestingly, PKA inhibition also reduces the phosphorylation levels of CREB, suggesting that PKA mediates genes activation during learning. This finding is in accord with our previous study demonstrating that motor learning induces genes expression [7]. The current proposed molecular model is that STEP activation acts as a tonic brake on synaptic transmission, which is associated with a decline in memory function [37]–[39]. Dephosphorylation of STEP increases its activity that will, in turn, inactivates key signaling molecules reinforcing synaptic plasticity, such as extracellular signal-regulated kinase 1/2 (ERK1/2) or NR2B subunit of *N*-methyl-_D_-aspartate receptors (NMDARs) [23], [40], [41]. In accord with this contention, STEP inhibition in CA1 hippocampal neurons enhanced transmission and occluded LTP induction [42]. Genetic deletion of STEP in mice enhanced spatial memory and fear conditioning consolidation [22]–[24]. Our data demonstrate that during motor learning, phosphorylation of STEP61 in the striatum is PKA dependant. According to these previous evidences, this finding is crucial to the rotarod learning and further demonstrates the importance of sustained inactivation of STEP during the learning of a complex motor skill.

We demonstrate that direct striatal infusion of a PKA inhibitor alters the acquisition of rotarod motor task. At high dose of Rp-cAMPS (40 µg/side), inhibition of PKA impairs considerably mice performances on the rotarod task. Our additional experiments looking at mice motor abilities suggest that this dose produces severe motor control deficits including bradykinesia, motor coordination troubles and akinesia, which are not observed at lower doses. Interestingly, however, when the rotarod task is fully learned, inhibition of PKA with 40 µg/side of Rp-cAMPS did not decreased rotarod performances. Once this particular task is learned, movements became automatically performed. This data suggest that PKA is not engage when movement is performed spontaneously, probably because these automatized movements do not require excessive skills or motor control. In mice lacking the RIIβ subunit of PKA, performances on the accelerating rotarod are slightly improved over two training days; but knockout mice never accomplish the same performances as wild type mice [43]. Even at a less challenging rate of acceleration, rotarod performances remain dramatically lower. In the striatum of these mice, PKA activity is reduced by 75%, which concur with our finding using high dose of PKA inhibitor. Our most compelling data are from the intermediate dose, 20 µg/side, for which treated-mice reached the maximal scores of performance at the fourth training day, whereas drug-naïve mice reach them at the last trials of the second day. Motor coordination and abilities are not altered at this dose. Therefore, unambiguously, the mice treated with 20 µg/side of Rp-cAMPS exhibited learning delays. The role of PKA in striatum-dependant motor learning paradigm is not clearly defined. Indirect evidence demonstrate that mice with genetic deletion of adenylyl cyclase 5, which is known to downregulate PKA activity through a reduction of cAMP levels, impairs the acquisition of striatum-dependant learning including response learning in the cross maze and motor skill learning associated with the accelerating rotarod [44]. We believe, based on these findings, that there is a ceiling effect of PKA inhibition in the striatum toward motor learning and that PKA inhibition is more vulnerable to motor skill leaning than motor control.

In conclusion, we report that attenuation of striatal STEP61 activity through PKA action is a crucial part of the molecular pathway leading to the automatization of a complex motor skill. Moreover, we highlight a regional brain variation in the levels of phosphorylated STEP61 at Ser221 during motor learning.

## Supporting Information

Figure S1 **STEP antibodies specificities.** (**A**) Representative examples of phospho-STEP61 and total STEP61 expression in the anterior cortex, hippocampus, striatum and cerebellum of mice. Brain lysates were immunoblotted with phospho-STEP and STEP total antibodies. According to the literature, STEP61 was observed in the anterior cortex, hippocampus and striatum, but not in the cerebellum. It is noteworthy that highest STEP61 expression levels were noticed in the striatum. The phospho-STEP61 antibody revealed that the lower band corresponded selectively to phosphorylated STEP61 at Ser221. (**B**) Specificity of the phospho-STEP antibody using alkaline phosphatase treatment on striatum lysates. Striatum lysates were incubated with or without phosphatase alkaline (calf intestinal). Protein extracts were immunoblotted with phospho-STEP antibody. No labelling was observed with the phospho-STEP antibody after phosphatase treatment. Reprobing the same membrane with total STEP antibody confirmed the presence of STEP61.
(TIF)

## References

[1] Ungerleider LG, Doyon J, Karni A (2002) Imaging brain plasticity during motor skill learning. Neurobiol Learn Mem 78: 553–564. 12559834 10.1006/nlme.2002.4091

[2] Doyon J, Bellec P, Amsel R, Penhune V, Monchi O, et al (2009) Contributions of the basal ganglia and functionally related brain structures to motor learning. Behav Brain Res 199: 61–75. 19061920 10.1016/j.bbr.2008.11.012

[3] Luft AR, Buitrago MM, Ringer T, Dichgans J, Schulz JB (2004) Motor skill learning depends on protein synthesis in motor cortex after training. J Neurosci 24: 6515–6520. 15269262 10.1523/JNEUROSCI.1034-04.2004 PMC6729880

[4] Willuhn I, Steiner H (2006) Motor-skill learning-associated gene regulation in the striatum: effects of cocaine. Neuropsychopharmacology 31: 2669–2682. 16395306 10.1038/sj.npp.1300995

[5] Wachter T, Rohrich S, Frank A, Molina-Luna K, Pekanovic A, et al (2010) Motor skill learning depends on protein synthesis in the dorsal striatum after training. Exp Brain Res 200: 319–323. 19823812 10.1007/s00221-009-2027-7

[6] Bureau G, Carrier M, Lebel M, Cyr M (2010) Intrastriatal inhibition of extracellular signal-regulated kinases impaired the consolidation phase of motor skill learning. Neurobiol Learn Mem 94: 107–115. 20447478 10.1016/j.nlm.2010.04.008

[7] D’Amours G, Bureau G, Boily MJ, Cyr M (2011) Differential gene expression profiling in the mouse brain during motor skill learning: focus on the striatum structure. Behav Brain Res 221: 108–117. 21376085 10.1016/j.bbr.2011.02.030

[8] Seeds NW, Williams BL, Bickford PC (1995) Tissue plasminogen activator induction in Purkinje neurons after cerebellar motor learning. Science 270: 1992–1994. 8533091 10.1126/science.270.5244.1992

[9] Kleim JA, Lussnig E, Schwarz ER, Comery TA, Greenough WT (1996) Synaptogenesis and Fos expression in the motor cortex of the adult rat after motor skill learning. J Neurosci 16: 4529–4535. 8699262 10.1523/JNEUROSCI.16-14-04529.1996 PMC6578852

[10] Kleim JA, Vij K, Ballard DH, Greenough WT (1997) Learning-dependent synaptic modifications in the cerebellar cortex of the adult rat persist for at least four weeks. J Neurosci 17: 717–721. 8987793 10.1523/JNEUROSCI.17-02-00717.1997 PMC6573226

[11] Meyer DA, Richer E, Benkovic SA, Hayashi K, Kansy JW, et al (2008) Striatal dysregulation of Cdk5 alters locomotor responses to cocaine, motor learning, and dendritic morphology. Proc Natl Acad Sci U S A 105: 18561–18566. 19017804 10.1073/pnas.0806078105 PMC2587606

[12] Lombroso PJ, Murdoch G, Lerner M (1991) Molecular characterization of a protein-tyrosine-phosphatase enriched in striatum. Proc Natl Acad Sci U S A 88: 7242–7246. 1714595 10.1073/pnas.88.16.7242 PMC52270

[13] Lombroso PJ, Naegele JR, Sharma E, Lerner M (1993) A protein tyrosine phosphatase expressed within dopaminoceptive neurons of the basal ganglia and related structures. J Neurosci 13: 3064–3074. 8331384 10.1523/JNEUROSCI.13-07-03064.1993 PMC6576687

[14] Boulanger LM, Lombroso PJ, Raghunathan A, During MJ, Wahle P, et al (1995) Cellular and molecular characterization of a brain-enriched protein tyrosine phosphatase. J Neurosci 15: 1532–1544. 7869116 10.1523/JNEUROSCI.15-02-01532.1995 PMC6577844

[15] Paul S, Snyder GL, Yokakura H, Picciotto MR, Nairn AC, et al (2000) The Dopamine/D1 receptor mediates the phosphorylation and inactivation of the protein tyrosine phosphatase STEP via a PKA-dependent pathway. J Neurosci 20: 5630–5638. 10908600 10.1523/JNEUROSCI.20-15-05630.2000 PMC6772528

[16] Abel T, Nguyen PV (2008) Regulation of hippocampus-dependent memory by cyclic AMP-dependent protein kinase. Prog Brain Res 169: 97–115. 18394470 10.1016/S0079-6123(07)00006-4 PMC2914307

[17] Abrams TW (2009) Cyclic AMP (cAMP) Role in Learning and Memory. In: Squire LR, editor. Encyclopedia of Neuroscience. Oxford: Academic press. 265–277.

[18] Schafe GE, LeDoux JE (2000) Memory consolidation of auditory pavlovian fear conditioning requires protein synthesis and protein kinase A in the amygdala. J Neurosci 20: RC96. 10974093 10.1523/JNEUROSCI.20-18-j0003.2000 PMC6772816

[19] Sharifzadeh M, Sharifzadeh K, Naghdi N, Ghahremani MH, Roghani A (2005) Posttraining intrahippocampal infusion of a protein kinase AII inhibitor impairs spatial memory retention in rats. J Neurosci Res 79: 392–400. 15622518 10.1002/jnr.20358

[20] Abel T, Nguyen PV, Barad M, Deuel TA, Kandel ER, et al (1997) Genetic demonstration of a role for PKA in the late phase of LTP and in hippocampus-based long-term memory. Cell 88: 615–626. 9054501 10.1016/s0092-8674(00)81904-2

[21] Isiegas C, Park A, Kandel ER, Abel T, Lattal KM (2006) Transgenic inhibition of neuronal protein kinase A activity facilitates fear extinction. J Neurosci 26: 12700–12707. 17151273 10.1523/JNEUROSCI.2743-06.2006 PMC2910919

[22] Paul S, Olausson P, Venkitaramani DV, Ruchkina I, Moran TD, et al (2007) The striatal-enriched protein tyrosine phosphatase gates long-term potentiation and fear memory in the lateral amygdala. Biol Psychiatry 61: 1049–1061. 17081505 10.1016/j.biopsych.2006.08.005 PMC1853327

[23] Venkitaramani DV, Moura PJ, Picciotto MR, Lombroso PJ (2011) Striatal-enriched protein tyrosine phosphatase (STEP) knockout mice have enhanced hippocampal memory. Eur J Neurosci 33: 2288–2298. 21501258 10.1111/j.1460-9568.2011.07687.x PMC3118976

[24] Olausson P, Venkitaramani DV, Moran TD, Salter MW, Taylor JR, et al (2012) The tyrosine phosphatase STEP constrains amygdala-dependent memory formation and neuroplasticity. Neuroscience 225: 1–8. 22885232 10.1016/j.neuroscience.2012.07.069 PMC3725644

[25] Baldwin AE, Sadeghian K, Holahan MR, Kelley AE (2002) Appetitive instrumental learning is impaired by inhibition of cAMP-dependent protein kinase within the nucleus accumbens. Neurobiol Learn Mem 77: 44–62. 11749085 10.1006/nlme.2000.4002

[26] Gerdjikov TV, Giles AC, Swain SN, Beninger RJ (2007) Nucleus accumbens PKA inhibition blocks acquisition but enhances expression of amphetamine-produced conditioned activity in rats. Psychopharmacology (Berl) 190: 65–72. 17047929 10.1007/s00213-006-0590-1

[27] Paxinos G, Francklin KBJ (2001) The mouse brain in stereotaxic coordinates: Academic Press.

[28] Chagniel L, Robitaille C, Lacharite-Mueller C, Bureau G, Cyr M (2012) Partial dopamine depletion in MPTP-treated mice differentially altered motor skill learning and action control. Behav Brain Res 228: 9–15. 22127145 10.1016/j.bbr.2011.11.019

[29] Lemay-Clermont J, Robitaille C, Auberson YP, Bureau G, Cyr M (2011) Blockade of NMDA receptors 2A subunit in the dorsal striatum impairs the learning of a complex motor skill. Behav Neurosci 125: 714–723. 21859173 10.1037/a0025213

[30] Palkovits M, Brownstein, M J. (1983) Microdissection of brain areas by the punch technique. In: Cuello AC, editor. Brain Microdissection Techniques. New York: Wiley. 1–36.

[31] Ogawa N, Mizukawa K, Hirose Y, Kajita S, Ohara S, et al (1987) MPTP-induced parkinsonian model in mice: biochemistry, pharmacology and behavior. Eur Neurol 26 Suppl 116–23. 2884111 10.1159/000116351

[32] Sedelis M, Hofele K, Auburger GW, Morgan S, Huston JP, et al (2000) MPTP susceptibility in the mouse: behavioral, neurochemical, and histological analysis of gender and strain differences. Behav Genet 30: 171–182. 11105391 10.1023/a:1001958023096

[33] Sedelis M, Schwarting RK, Huston JP (2001) Behavioral phenotyping of the MPTP mouse model of Parkinson’s disease. Behav Brain Res 125: 109–125. 11682102 10.1016/s0166-4328(01)00309-6

[34] Blume SR, Cass DK, Tseng KY (2009) Stepping test in mice: a reliable approach in determining forelimb akinesia in MPTP-induced Parkinsonism. Exp Neurol 219: 208–211. 19460369 10.1016/j.expneurol.2009.05.017

[35] Lebel M, Patenaude C, Allyson J, Massicotte G, Cyr M (2009) Dopamine D1 receptor activation induces tau phosphorylation via cdk5 and GSK3 signaling pathways. Neuropharmacology 57: 392–402. 19591849 10.1016/j.neuropharm.2009.06.041

[36] Kleim JA, Hogg TM, VandenBerg PM, Cooper NR, Bruneau R, et al (2004) Cortical synaptogenesis and motor map reorganization occur during late, but not early, phase of motor skill learning. J Neurosci 24: 628–633. 14736848 10.1523/JNEUROSCI.3440-03.2004 PMC6729261

[37] Braithwaite SP, Paul S, Nairn AC, Lombroso PJ (2006) Synaptic plasticity: one STEP at a time. Trends Neurosci 29: 452–458. 16806510 10.1016/j.tins.2006.06.007 PMC1630769

[38] Fitzpatrick CJ, Lombroso PJ (2011) The Role of Striatal-Enriched Protein Tyrosine Phosphatase (STEP) in Cognition. Front Neuroanat 5: 47. 21863137 10.3389/fnana.2011.00047 PMC3149150

[39] Goebel-Goody SM, Baum M, Paspalas CD, Fernandez SM, Carty NC, et al (2012) Therapeutic implications for striatal-enriched protein tyrosine phosphatase (STEP) in neuropsychiatric disorders. Pharmacol Rev 64: 65–87. 22090472 10.1124/pr.110.003053 PMC3250079

[40] Paul S, Nairn AC, Wang P, Lombroso PJ (2003) NMDA-mediated activation of the tyrosine phosphatase STEP regulates the duration of ERK signaling. Nat Neurosci 6: 34–42. 12483215 10.1038/nn989

[41] Braithwaite SP, Adkisson M, Leung J, Nava A, Masterson B, et al (2006) Regulation of NMDA receptor trafficking and function by striatal-enriched tyrosine phosphatase (STEP). Eur J Neurosci 23: 2847–2856. 16819973 10.1111/j.1460-9568.2006.04837.x

[42] Pelkey KA, Askalan R, Paul S, Kalia LV, Nguyen TH, et al (2002) Tyrosine phosphatase STEP is a tonic brake on induction of long-term potentiation. Neuron 34: 127–138. 11931747 10.1016/s0896-6273(02)00633-5

[43] Brandon EP, Logue SF, Adams MR, Qi M, Sullivan SP, et al (1998) Defective motor behavior and neural gene expression in RIIbeta-protein kinase A mutant mice. J Neurosci 18: 3639–3649. 9570795 10.1523/JNEUROSCI.18-10-03639.1998 PMC6793128

[44] Kheirbek MA, Britt JP, Beeler JA, Ishikawa Y, McGehee DS, et al (2009) Adenylyl cyclase type 5 contributes to corticostriatal plasticity and striatum-dependent learning. J Neurosci 29: 12115–12124. 19793969 10.1523/JNEUROSCI.3343-09.2009 PMC2782774

