# Correction: Regulation of Tyrosine Phosphatase STEP61 by Protein Kinase A during Motor Skill Learning in Mice

**DOI:** 10.1371/journal.pone.0150220

**Published:** 2016-03-09

**Authors:** Laure Chagniel, Yan Bergeron, Geneviève Bureau, Guy Massicotte, Michel Cyr

The authors would like to correct Fig 2, as errors were introduced in the preparation of this figure for publication. The GAPDH panel shown in Fig 2C (Anterior Cortex) is incorrectly duplicated in the GADPH panel shown in Fig 2D (Striatum). The authors have provided a corrected Fig 2D here. The authors confirm that these changes do not alter their findings. The raw blot images are provided as a Supporting Information file.

**Fig 2:**
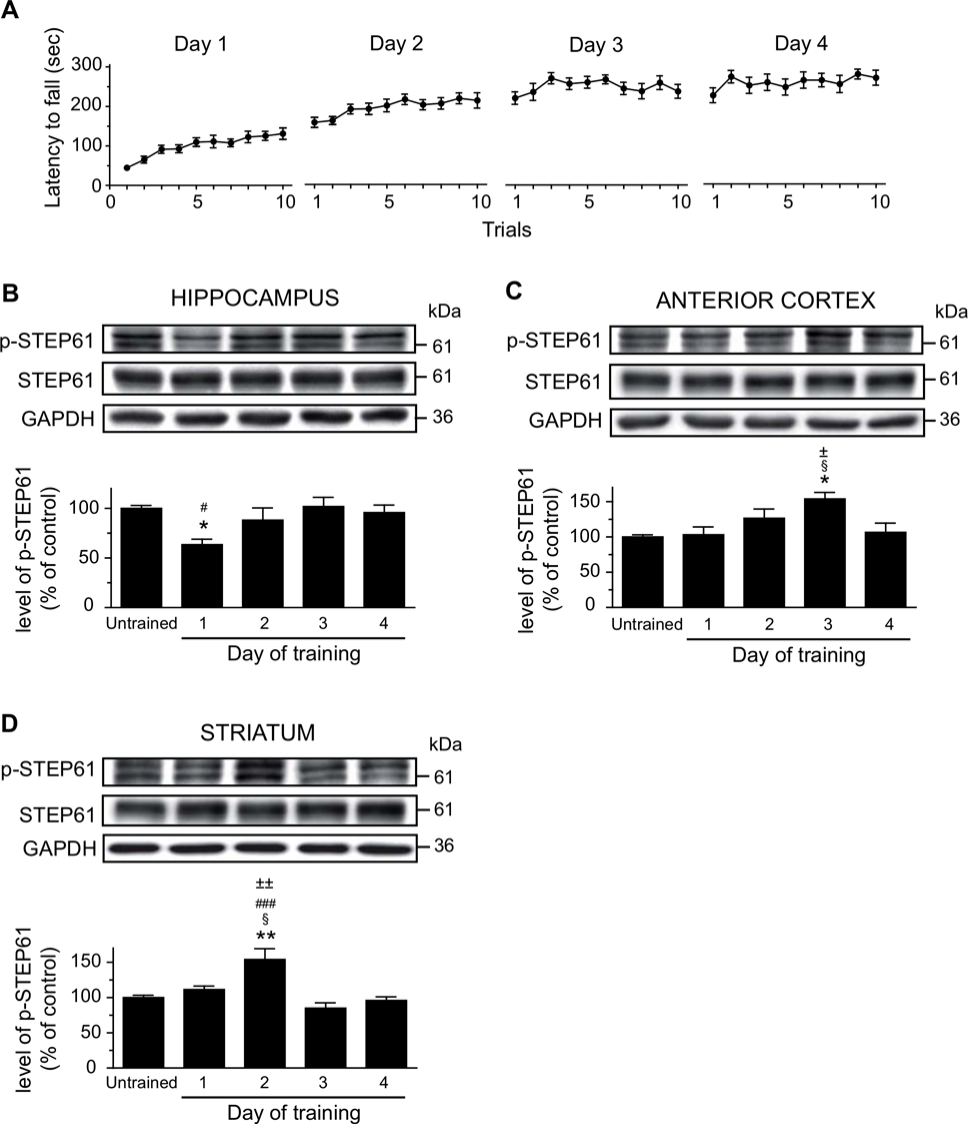
Levels of phosphorylated STEP61 in the brain of mice during motor learning. (A) Drug-naïve mice were trained on the accelerating rotarod during 4 consecutive days and sacrificed at the end of each training day. Protein levels were evaluated by western blot. Proteins were extracted from selected brain regions of untrained or trained mice. Protein levels of phosphorylated STEP61 at Ser221, total STEP61 as well as GAPDH were measured in (B) hippocampus, (C) anterior cortex and (D) striatum. Data represent the mean of p-STEP61 relative optical density (expressed as a percentage of control values) ± S.E.M. Values are expressed relative to total STEP and are from triplicate experiments/animal, n = 4 mice/group. *p<0.05, **p<0.01 vs. untrained mice; §p<0.05 vs day 1; #p<0.05, ###p<0.001 vs. day 3; ±p<0.05, ±±p<0.01 vs. day 4.

## Supporting Information

S1 File Uncropped blots. (PDF)
