# Supplementary figures and images for: Regulation of Tyrosine Phosphatase STEP61 by Protein Kinase A during Motor Skill Learning in Mice
Source: PLoS One. 2014 Jan 23;9(1):e86988. doi: 10.1371/journal.pone.0086988 (PMC3900697; doi:10.1371/journal.pone.0086988)

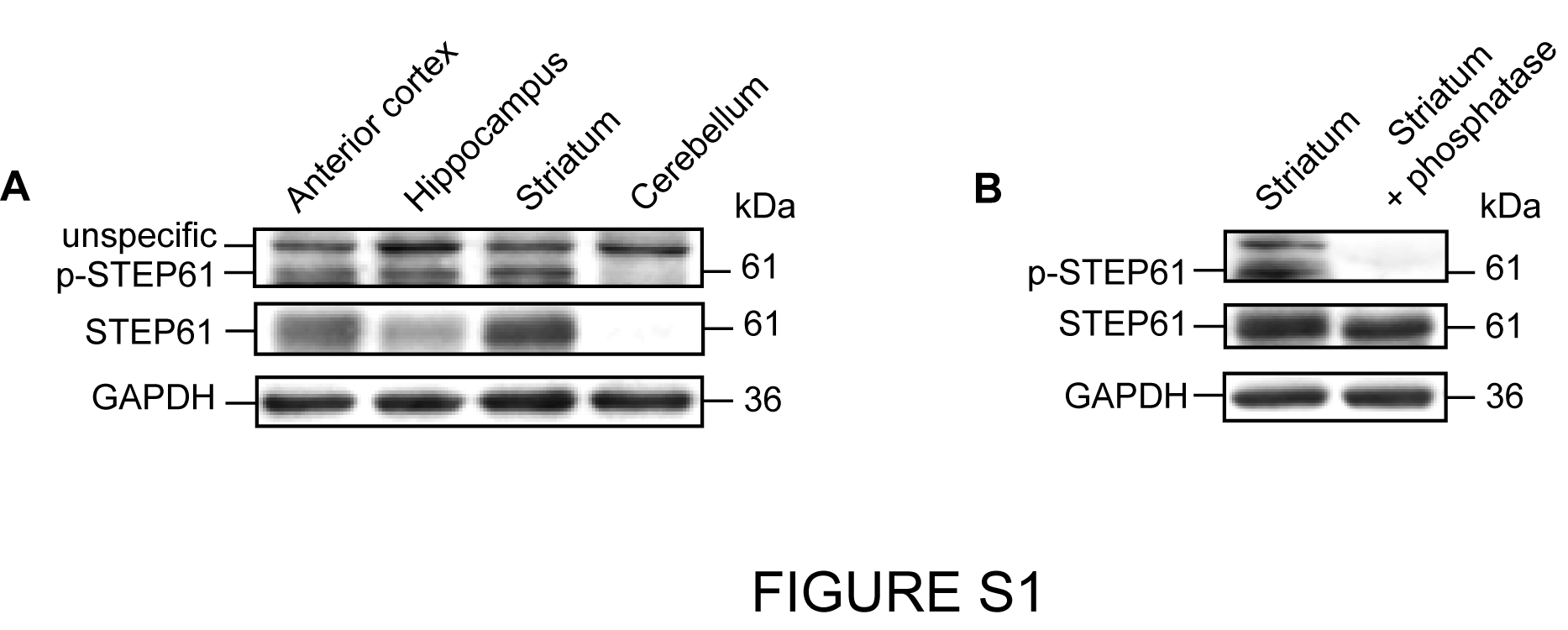

Supplement: Figure S1 — STEP antibodies specificities. (A) Representative examples of phospho-STEP61 and total STEP61 expression in the anterior cortex, hippocampus, striatum and cerebellum of mice. Brain lysates were immunoblotted with phospho-STEP and STEP total antibodies. According to the literature, STEP61 was observed in the anterior cortex, hippocampus and striatum, but not in the cerebellum. It is noteworthy that highest STEP61 expression levels were noticed in the striatum. The phospho-STEP61 antibody revealed that the lower band corresponded selectively to phosphorylated STEP61 at Ser221. (B) Specificity of the phospho-STEP antibody using alkaline phosphatase treatment on striatum lysates. Striatum lysates were incubated with or without phosphatase alkaline (calf intestinal). Protein extracts were immunoblotted with phospho-STEP antibody. No labelling was observed with the phospho-STEP antibody after phosphatase treatment. Reprobing the same membrane with total STEP antibody confirmed the presence of STEP61. (TIF) [file pone.0086988.s001.tif]
