# Supplementary figures and images for: Correction: Regulation of Tyrosine Phosphatase STEP61 by Protein Kinase A during Motor Skill Learning in Mice
Source: PLoS One. 2016 Mar 9;11(3):e0150220. doi: 10.1371/journal.pone.0150220 (PMC4784932; doi:10.1371/journal.pone.0150220)

Raw blots of GAPDH for each triplicate/ n

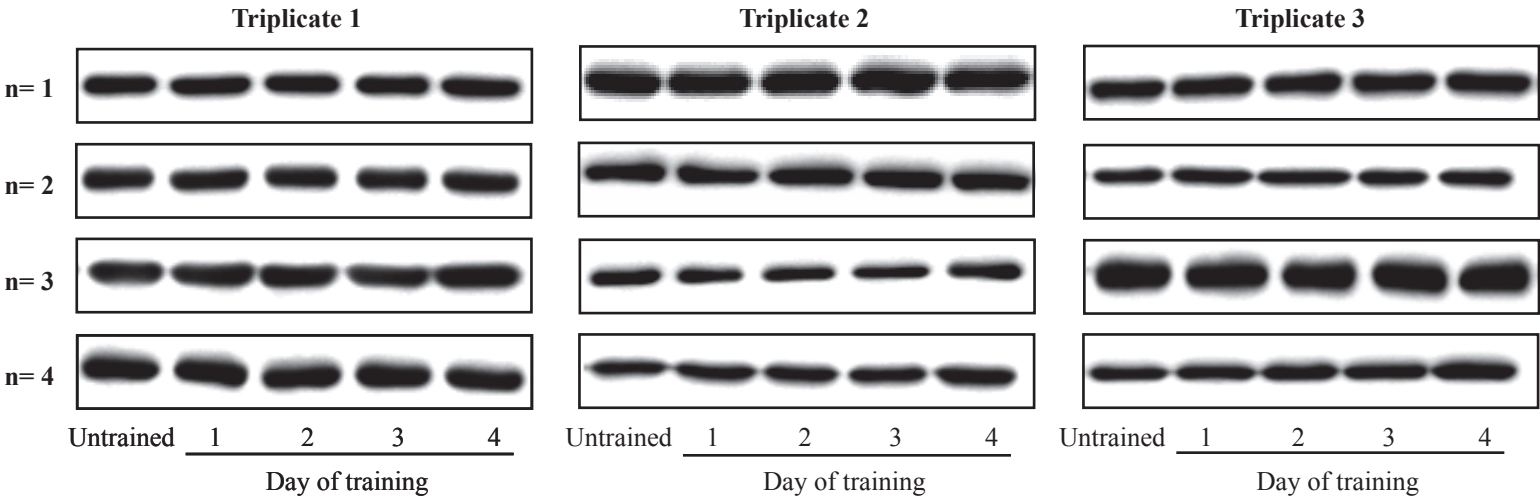

Supplement: S1 File — (PDF) [file pone.0150220.s001.pdf]
